# How are ontologies implemented to represent clinical practice guidelines in clinical decision support systems: protocol for a systematic review

**DOI:** 10.1186/s13643-022-02063-7

**Published:** 2022-08-31

**Authors:** Fatemeh Sadeghi-Ghyassi, Shahla Damanabi, Leila R. Kalankesh, Stijn Van de Velde, Mohammad-Reza Feizi-Derakhshi, Sakineh Hajebrahimi

**Affiliations:** 1 Department of Health Information Technology, School of Management and Medical Informatics, Tabriz University of Medical Sciences, Tabriz, Iran; 2 Research Center for Evidence Based-Medicine, Iranian EBM Center: A Joanna Briggs Institute Center of Excellence, Tabriz University of Medical Sciences, Tabriz, Iran; 3 Health Services Management Research Center, School of Management and Medical Informatics, Tabriz University of Medical Sciences, Tabriz, Iran; 4 Norwegian Institute of Public Health, Oslo, Norway; 5 ComInSys Lab., Department of Computer Engineering, University of Tabriz, Tabriz, Iran

**Keywords:** Clinical decision support systems, Ontology, Biological ontologies, Semantics, Knowledge-based systems, Clinical practice guidelines, Computer-interpretable guidelines, Computer-assisted decision making

## Abstract

**Background:**

Clinical practice guidelines are statements which are based on the best available evidence, and their goal is to improve the quality of patient care. Integrating clinical practice guidelines into computer systems can help physicians reduce medical errors and help them to have the best possible practice. Guideline-based clinical decision support systems play a significant role in supporting physicians in their decisions. Meantime, system errors are the most critical concerns in designing decision support systems that can affect their performance and efficacy. A well-developed ontology can be helpful in this matter. The proposed systematic review will specify the methods, components, language of rules, and evaluation methods of current ontology-driven guideline-based clinical decision support systems.

**Methods:**

This review will identify literature through searching MEDLINE (via Ovid), PubMed, EMBASE, Cochrane Library, CINAHL, ScienceDirect, IEEEXplore, and ACM Digital Library. Gray literature, reference lists, and citing articles of the included studies will be searched. The quality of the included studies will be assessed by the mixed methods appraisal tool (MMAT-version 2018). At least two independent reviewers will perform the screening, quality assessment, and data extraction. A third reviewer will resolve any disagreements. Proper data analysis will be performed based on the type of system and ontology engineering evaluation data.

**Discussion:**

The study will provide evidence regarding applying ontologies in guideline-based clinical decision support systems. The findings of this systematic review will be a guide for decision support system designers and developers, technologists, system providers, policymakers, and stakeholders. Ontology builders can use the information in this review to build well-structured ontologies for personalized medicine.

**Systematic review registration:**

PROSPERO CRD42018106501

**Supplementary Information:**

The online version contains supplementary material available at 10.1186/s13643-022-02063-7.

## Background

The role of clinical decision support systems (CDSS) in helping clinicians to make decisions is becoming increasingly important. CDSSs are computer-based systems that augment clinicians in decision-making throughout patient care [1]. According to EBHC pyramid 5.0 for accessing pre-appraised evidence, CDSSs rest at the top of the pyramid, and they are designed based on information sources such as clinical practice guidelines (CPG), systematic reviews, and evidence summaries [2]. The literature shows the potential positive impact of CDSSs on physicians’ decision-making [3–7]. However, a recent meta-analysis shows that the pooled impact of CDSSs on guideline adherence during patient care is relatively low [8]. Given the positive and negative effects of decision support systems in clinical practice, it is necessary to investigate the causes of the success and failure of CDSSs in helping to improve the treatment of patients. To achieve this goal, using a well-developed checklist to understand better the concepts and components of a valuable and compatible system can benefit both system developers and end-users. The GUIDES checklist is one of the valuable tools that provide a correct and detailed understanding of the factors that contribute to the effectiveness of guideline-based CDSSs [9]. High-quality patient data is one of the critical factors identified in the GUIDES checklist. This factor relies on the use of appropriate data systems, standards, and terminologies. In literature, there are multiple categories for CDSSs. From a perspective, CDSSs can fall into knowledge-based and nonknowledge-based systems [1]. Nonknowledge-based CDSSs learn from past experiences and are based on pattern recognition [1]. They use machine learning to achieve this purpose. In contrast, the primary purpose of a knowledge-based decision support system is to make the best and accurate decision based on the most reliable knowledge. CPGs are known as evidence-based resources in medicine that can be a reliable source of knowledge for a knowledge-based CDSS. In recent decades, translating the knowledge contained in a CPG into practice in the form of a knowledge-based system is attracting more attention from systems designers, experts, and stakeholders in healthcare. In knowledge-based systems, the representation of the knowledge is crucial. For making the best decision by CDSSs, utilization of the best evidence is essential but not enough. For an acceptable performance and minimum defect, we need specific efficient auxiliary tools to be applied in a decision support system when designing the system. Ontologies are one of the tools for knowledge representation in CDSSs. An ontology provides a formal approach for representing the concepts used in the domain knowledge and their relations. At the same time, ontology provides interoperability and easy sharing of knowledge. Presenting the knowledge in a decision support system by ontology makes integrating structured knowledge and data possible [10]. Ontologies make it possible to represent concepts semantically in the system. This sort of representation can be helpful in the whole process of decision-making, ranging from the phase of data collection from different heterogeneous data sources (data integration) to automating logical reasoning of the inference steps of the DSS decision-making process such as ontology reasoners [10, 11]. Developing an ontology based on a CPG makes it possible to convert unstructured information contained in the guideline into structured knowledge. Accordingly, the information contained in the guideline is extracted in the form of concepts, text words, and their synonyms. Then, the properties and features of the concepts as well as the relationship between the concepts are determined. Finally, the CPG ontology is hierarchically structured in terms of concepts, attributes, relations, and instances. Different methods are used to convert the information of CPGs into ontologies, each with its own characteristics and applications. Also, the type, number of various guidelines, the method of using guidelines’ knowledge, and recommendations in the construction of rules, the combination of other methods of artificial intelligence in the development of ontology-based systems can be varied.

Recently, ontological reasoning has been widely used in many decision support systems, including CDSSs. With ontological reasoning, new facts that are not explicitly stated in the ontology are inferred and discovered. Various studies have explained approaches for adopting ontologies in CDSSs [12–17]. Evaluating and understanding the technical factors involved in building effective ontology-based CDSSs can pave the way for more efficient system design and selection. Several systematic literature reviews have been conducted on implementing ontologies on information systems and technology [18–22]. However, as far as we know, no systematic review has specifically reviewed the implementation of ontologies in guideline-based clinical decision support systems. It should be noted that in some reviews, guideline-based CDSSs were part of the studies included in the review [22]. In the present protocol, the technical characteristics of using guidelines in the construction of ontologies and systems will be assessed in more depth. In addition, more bibliographic databases and resources will be searched. The quality of included studies will be assessed by a validated appraisal tool. Also, the search date coverage will cover recent studies that have not been included in previous systematic reviews. The primary aim of this review is to determine the ontology design methods, ontology components, system architecture, features of the guidelines, language of rules, and evaluation methods of current ontology-driven guideline-based CDSSs. The secondary objective is to assess how ontologies could potentially impact improving decision-making in guideline-based CDSSs.

## Methods/design

This review tries to answer these questions:

1. What are the ontological approaches, design methods, and components of ontology-driven guideline-based CDSSs?
2. What languages have been used for constructing rules of ontology-driven guideline-based CDSSs?
3. How have the ontology and ontology-driven guideline-based CDSSs been evaluated?
4. How can ontological reasoning improve guideline-based decision-making as appraised by experts?

The protocol for this systematic review is registered on the International Prospective Register of Systematic Reviews (PROSPERO) as CRD 42018106501 and is available on the University of York website [23]. The Preferred Reporting Items for Systematic Reviews and Meta-Analysis Protocols (PRISMA-P) checklist has been used to prepare this protocol [24]. A completed PRISMA-P checklist is attached as Additional file 1. The systematic review will be reported following the Preferred Reporting Items for Systematic Reviews and Meta-Analyses (PRISMA) statement.

### Study eligibility criteria

Table 1 presents the critical research elements in the PO frame. The review will include all studies that report the development and implementation of an ontology in the structure and architecture of a guideline-based CDSS, using quantitative or qualitative, or mixed methods. The review will exclude any studies that report gene ontologies in DSSs and nonknowledge-based CDSSs. Reviews, editorials, letters to editors, commentary, and opinion pieces will be excluded as well. There will be no language and publication date restrictions.

**Table 1:** The main study elements in PO format

	Description
Population	Any guideline-oriented ontological decision support system
outcome	*Primary outcome(s):* Specifications of ontologies ontology design methods Specifications of the guidelines used Computer-interpretable guidelines (CIGs) models Language of rules System architecture Ontology evaluation methods System evaluation methods *Secondary outcome(s):* Effect of ontologies on potential to improve guideline-based decision making as appraised by experts

### Search strategy

The electronic databases that will be searched include but are not limited to MEDLINE (via Ovid), PubMed, Embase, Cochrane Library, CINAHL (via EBSCO), ScienceDirect, ProQuest Dissertations & Theses, IEEE Xplore, ACM Digital Library, and AISeL. It should be noted that since MEDLINE search in Ovid and PubMed platforms often leads to retrieving slightly different results due to their different search algorithms, we preferred to search both platforms in order not to miss any possible related studies. Additional searches will be done on Google Scholar for any possible studies. The search strategy piloted initially with a broad pattern and refined according to the systematic review aims and PO frame.

Gray literature, reference lists and citing articles of the included studies, and reference lists of published reviews in the subject will be tracked. The authors of articles published with incomplete information will be contacted. The key search terms include but are not limited to ontology, clinical practice guidelines, and clinical decision support systems. The search strategy will include free text and subject headings (controlled vocabularies) related to selected keywords. A draft search strategy of MEDLINE is available in Additional file 2. The search strategy will be adapted according to the other databases’ search options.

### Study selection

All the retrieved citations will be imported into Covidence systematic review management software after removing duplicates. Screening of the studies, data extraction, and quality assessment will be done via Covidence. Based on inclusion and exclusion criteria, two independent reviewers will review the titles and abstracts of retrieved studies. Then, they will review the full text of selected studies to determine their eligibility and consult with the third reviewer to resolve any disagreements. The reasons for the exclusion of any assessed article will be documented. The flow of the study selection process will be reported using a PRISMA 2020 study flow diagram [25].

### Quality assessment

Two reviewers will independently assess the quality of included studies. A third reviewer will be available in the case of disagreement. Because we will conduct a review of mixed-method studies, the quality of the included studies will be assessed by the mixed-method appraisal tool (MMAT-version 2018) [26]. The MMAT is a critical appraisal tool designed to appraise the methodological quality of five categories to studies: qualitative research, randomized controlled trials, non-randomized studies, quantitative descriptive studies, and mixed methods studies [26]. Scores of the MMAT vary from 25 to 100% (according to study type criteria). Studies will not be excluded by quality assessment, but lower quality studies will be reviewed to determine if they affect the study results.

### Data extraction

All reviewers will be involved in extracting the data. To assess the validity and reliability of the data extraction form, the researchers will pilot it on 10% of included studies to apply necessary changes. The researchers will organize data in the following categories:

- Characteristics of the studies (e.g., publication year, authors, study type, country of study)
- Reported findings of the system (e.g., setting, ontology development method, ontology development tool, ontology language, phase of system development, system architecture, type of implementation, the language of rules, CDSS target user, system’s outcome)
- Reported findings of guidelines (e.g., the title of the guideline(s), the scope of the guideline, guideline-based rules, original/adapted/adopted guidelines, number of guidelines used, and computer-interpretable guidelines (CIGs) models)
  - Methods of ontology evaluations
  - Methods of the system evaluation
  - Primary and secondary outcome measures and the quality assessment scores using the MMAT appraisal tool

To achieve the secondary objective, the opinions of experts and specialists will be collected on the effectiveness of guideline-oriented ontological CDSSs. The data will be extracted from the eligible studies in which experts’ opinions have been reported in the form of questionnaires or qualitative study themes to achieve a consensus.

If any disagreements arise between the reviewers, these will be resolved through discussion.

### Data synthesis

Because of the variation among the studies, based on the results, a narrative review will be conducted to describe the key characteristics, components, and any similarities in included studies to define how results could be encapsulated [27]. Additional data analysis will be performed if required based on the type of system and ontology engineering evaluation data, and experts’ opinions on the effectiveness of CDSSs. We will not perform a meta-analysis.

## Discussion

By conducting this review, we will provide a comprehensive overview of applying ontology reasoning in guideline-based clinical decision support systems. System architecture and ontology design are fundamental in ontology-driven guideline-based CDSS performance. The framework of ontology-driven CDSS needs persistent modification and improvement to lead to a trusted and evidence-based decisions. Using ontologies can be beneficial in content maintenance of decision support systems which is one of the challenges of knowledge-based systems [28].

There have been few reviews of reasoning methodologies, including ontology reasoning in CDSSs [22, 29, 30]. However, this review specifically and systematically will assess ontology reasoning in guideline-based decision support systems. This systematic review will provide evidence regarding adopting ontology in guideline-based CDSSs. We expect that our findings will guide decision support system designers and developers, technologists, system providers, policymakers, and stakeholders. We anticipate that ontology builders can use the information in this review to build well-structured and functional biological and clinical ontologies for utilization in personalized medicine. Also, the findings of this study can be used in the development and improvement of tools for evaluating ontology-driven decision support systems.

This review will have some strengths. First, the review will have a systematic and comprehensive search in medical and computer science black and gray literature. Second, the review will extract and summarize all aspects of ontology building, evaluation, and employment in guideline-based CDSSs. The limitation of the study is that some of the included studies are prototype systems, and the designed systems are not set up and implemented in a natural clinical setting.

## Supplementary Information

**Additional file 1.** PRISMA 2015 checklist

**Additional file 2.** Search strategy MEDLINE

## Data Availability

Data are available upon request from the authors.

## Abbreviations

CDSS: Clinical Decision Support System
CIG: Computer-Interpretable Guideline
CPG: Clinical Practice Guidelines
PRISMA: Preferred Reporting Items for Systematic Reviews and Meta-Analyses
PRISMA-P: Preferred Reporting Items for Systematic Reviews and Meta-Analyses Protocols
PROSPERO: International Prospective Register of Systematic Reviews
