# Supplementary material for: How are ontologies implemented to represent clinical practice guidelines in clinical decision support systems: protocol for a systematic review
Source: Syst Rev. 2022 Aug 31;11:183. doi: 10.1186/s13643-022-02063-7 (PMC9429575; doi:10.1186/s13643-022-02063-7)
Supplement: Supplementary file 2 — Additional file 2. Search strategy MEDLINE [file 13643_2022_2063_MOESM2_ESM.doc]

**Additional file 2**

Database: Ovid MEDLINE(R) ALL / PubMed(R) <1946 to Present>

Search Strategy:

--------------------------------------------------------------------------------

1 exp Decision Support Systems, Clinical/

2 exp Medical Order Entry Systems/

3 exp Decision Making, Computer-Assisted/

4 exp Clinical Decision Rules/

5 (decision adj3 support*).ab,hw,kf,kw,ti.

6 (CDSS or MDSS).ab,hw,kf,kw,ti.

7 1 or 2 or 3 or 4 or 5 or 6

8 Biological Ontologies/

9 Semantics/

10 ontolog*.ab,hw,kf,kw,ti.

11 Semantic*.ab,hw,kf,kw,ti.

12 7 or 8 or 9 or 10 or 11

13 exp Practice Guidelines as Topic/

14 guideline*.ab,hw,kf,kw,ti.

15 13 or 14

16 7 and 12 and 15

***************************
